# Overcoming the Catalytic Bucket Effect in Pt‐based High‐Entropy Nanocages Through Interface Defect and Strain Engineering

**DOI:** 10.1002/advs.76501

**Published:** 2026-07-08

**Authors:** Qian Liu, Haoran Kang, Yiou Liu, Xiaowei Zhang, Xu Chen, Faming Gao

**Affiliations:** ^1^ Tianjin Key Laboratory of Multiplexed Identification For Port Hazardous Chemicals State Key Laboratory of Bio‐based Fiber Materials Tianjin University of Science & Technology Tianjin P. R. China; ^2^ Hebei Key Laboratory of Applied Chemistry Yanshan University Qinhuangdao P. R. China

**Keywords:** catalytic bucket effect, defect, fuel cells, oxygen reduction catalysts, Pt‐based high‐entropy nanocages, strain engineering

## Abstract

We demonstrate the fabrication of PtPdNiCuMnAu high‐entropy nanocages (HENCs) through a facile liquid‐phase reduction method followed by acid etching treatment. The multi‐component characteristic induces electron redistribution, which regulates the Pt *d*‐band center with effect. The incorporation of Mn, endowed with distinct atomic size and coordination environment, introduces significant lattice distortion and atomic‐level defects. Trace Au plays a crucial role in elevating the vacancy formation energy, thus significantly suppressing elemental segregation under electrochemical bias. Additionally, the acid etching treatment further exposes abundant active sites as well as precisely tailors the adsorption kinetics of oxygen intermediates. Benefiting from these synergistic effects, PtPdNiCuMnAu HENCs exhibit a remarkable half‐wave potential (*E*_1/2_) of 0.925 V *vs*. RHE and a high mass activity (MA) of 1.70 A/mg_Pt_. Furthermore, the catalyst displays exceptional electrochemical stability, with only a 4 mV negative shift in *E*_1/2_ and a mere 5.6% loss in MA after 10, 000 accelerated durability test cycles. With this catalyst integrated, the membrane electrode assembly demonstrates a remarkable peak power density of 381.6 ± 3.10 mW/cm^2^ under H_2_‐O_2_ conditions, achieved at a back pressure of 2.0 bar and a cathode loading of 0.2 mg_Pt_/cm^2^.

## Graphical abstract

PtPdNiCuMnAu HENCs exhibit a remarkable half‐wave potential (*E*_1/2_) of 0.925 V *vs*. RHE. The catalyst displays exceptional electrochemical stability, with only a 4 mV negative shift in *E*_1/2_ and a mere 5.6% loss in MA after 10, 000 accelerated durability test cycles.

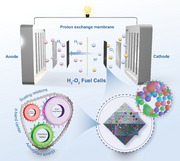

## 1 Introduction

Proton exchange membrane fuel cells (PEMFCs) have been widely recognized as a pivotal technology for hydrogen energy utilization, providing a sustainable pathway to achieve the dual goals of carbon neutrality and peak carbon emissions [1, 2, 3]. Nevertheless, the limited efficiency of PEMFCs largely stems from the slow rate of the cathodic oxygen reduction reaction (ORR) [4, 5]. Pt‐based alloys have been regarded as state‐of‐the‐art ORR electrocatalysts [6, 7, 8, 9], such as Toyota Mirai fuel cell vehicles [10]. Alloying Pt with transition metals (M) can modulate the electronic structure of key components through valence orbital interactions [11], further facilitating the adsorption/desorption behavior of oxygen‐containing intermediates [12, 13]. However, under the harsh operating conditions of PEMFCs (high voltage: 0.6–1.0 V, strong acidity: pH < 1), the inevitable dissolution and segregation of M lead to severe degradation in both catalytic activity and long‐term stability [14, 15].

Recently, high‐entropy alloy (HEA) nanomaterials, which comprise at least five elements forming a solid solution, have garnered considerable interest as high‐performance ORR electrocatalysts [16, 17]. These materials elevate the configurational entropy with the intention to minimize Gibbs free energy (Δ*G* = Δ*H*‐*T*Δ*S*) by stabilizing the strength of the system [18]. Their unique properties, including lattice distortion, high configurational entropy, synergistic effect among multiple elements, and sluggish atomic diffusion, contribute to exceptional functional performance [19]. The so‐called “cocktail effect” in HEAs implies that unexpected synergistic properties can be finely tuned by incorporating some specific elements, thereby facilitating the rational design of high‐efficiency catalysts [20]. Moreover, the disordered surfaces of HEAs (e.g., atomic vacancies and grain boundaries) can introduce micro‐strain [21, 22], a key descriptor that has been increasingly recognized to regulate ORR performance by optimizing intermediate adsorption behavior [23, 24]. Such a surface structure provides various nearby adsorption sites, making it efficient for ORR involving four‐electron transport [25]. Acid etching is commonly applied to generate more defective sites, refine surface micro‐strain, and reinforce the stability of Pt‐based alloy catalysts [22, 26, 27]. Additionally, reducing the material size increases the proportion of surface atoms, leading to improved mass activity and kinetic characteristics [28, 29, 30]. Nevertheless, the complex atomic coordination and relatively weak metal bonds in HEAs render them susceptible to element segregation and dissolution, analogous to the “bucket effect”, where the least stable component dictates overall durability. To date, the detailed mechanism underlying this bucket effect—specifically, the dynamic interplay between catalytic activity and electrochemical stability in HEAs—remains largely unexplored.

Herein, we synthesized PtPdNiCuMnAu high‐entropy nanocages (HENCs) via a facile liquid‐phase reduction method in an oil bath followed by acid etching, aiming to engineer a defective and strained interface through the synergistic “cocktail effect” of multi‐element composition and post‐treatment. The incorporation of Mn and Au was strategically designed: Mn, with its distinct ionic radius and coordination preference, is expected to introduce significant lattice distortion and atomic defects; while Au, with high electronegativity and vacancy formation energy, can modulate the electronic structure and suppress elemental segregation. The acid etching step further amplifies structural defects and lattice strain, synergistically optimizing the exposure of active sites and the adsorption behavior of oxygen‐containing intermediates. Systematic characterizations (e.g., HAADF‐STEM, XRD, XPS, CO‐TPD) and electrochemical measurements demonstrate that PtPdNiCuMnAu HENCs exhibit a highly disordered atomic arrangement, enlarged specific surface area, and tailored electronic structure. The as‐prepared materials possess remarkable structural stability via in situ heating transmission electron microscopy and accelerated durability tests. Density functional theory (DFT) calculations reveal that the downshifted Pt *d*‐band center—induced by Mn/Au incorporation and lattice strain—optimizes the binding strength of oxygen‐containing intermediates, thereby accelerating ORR kinetics. Notably, PtPdNiCuMnAu HENCs achieve a half‐wave potential (*E*_1/2_) of 0.925 V *vs*. RHE and a mass activity of 1.70 A/mg_Pt_ at 0.9 V *vs*. RHE, which is 2.79 and 7.39 times higher than those of PtPdNiCu NCs and commercial Pt/C, respectively. The catalyst demonstrates exceptional stability, as evidenced by a minimal loss of only 4 mV in *E*_1/2_ and a mere 5.6% degradation in mass activity after 10 000 cycles. When integrated into a membrane electrode assembly, this catalyst yields a peak power density of 319.7 ± 3.36 mW cm^−2^ and 381.6 ± 3.10 mW cm^−2^ under different back pressures, respectively. This outstanding activity and remarkable durability are attributed to comprehensive effects of entropy stabilization, micro‐strain engineering, and suppressed elemental segregation.

## 2 Results and Discussion

**FIGURE 1:**
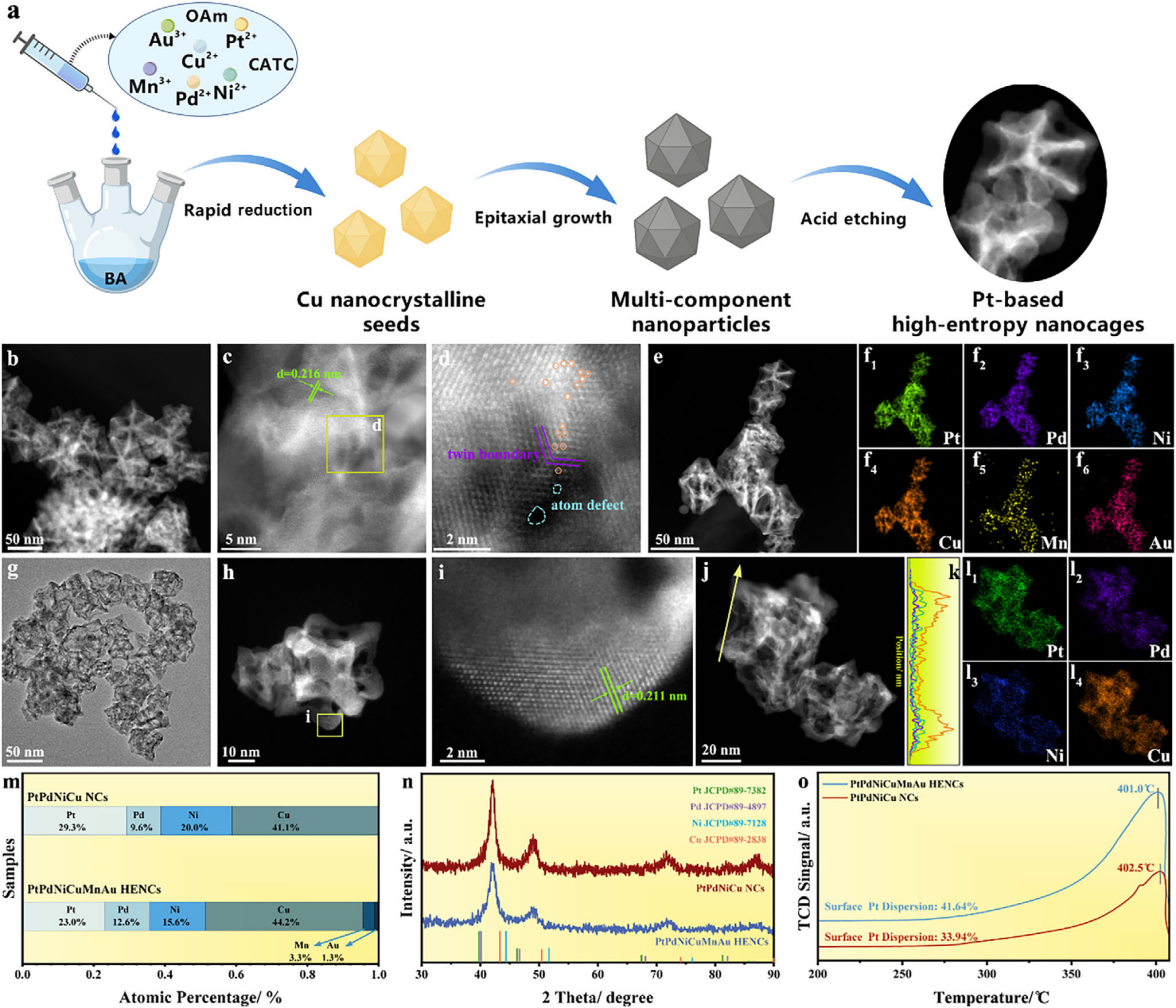
(a) Schematic illustration of the liquid‐phase reduction process to synthesize PtPdNiCuMnAu HENCs. (b‐e) HAADF‐STEM images and (f_1_‐f_6_) corresponding elemental mapping of PtPdNiCuMnAu HENCs. (g) TEM image, (h‐j) HAADF‐STEM images, and (k, l_1_‐l_4_) corresponding elemental line scan and mapping of PtPdNiCu NCs. (m) The elemental composition from ICP‐OES results, (n) XRD patterns, and (o) CO‐TPD curves of PtPdNiCuMnAu HENCs and PtPdNiCu NCs.

The schematic in Figure 1a illustrates the synthesis process of PtPdNiCuMnAu high‐entropy nanocages (HENCs). Initially, Pt‐based alloy nanoparticles were prepared via a liquid‐phase reduction method. Notably, the dropwise addition strategy was employed instead of the classic one‐pot direct heating method. This approach effectively balances the reduction rates of each component and optimizes coordination effects. Specifically, the drip strategy accelerates the relative reduction rate of Ni. As a result, the Ni content reached 33.9% under the drip strategy while only 0.49% under the one‐pot strategy with the same reaction time (Figure S1 and Table S1). Moreover, under the one‐pot strategy, Pd tends to nucleate rapidly and distribute internally [31], forming a Pd‐core structure which is unfavorable for constructing hollow structures with higher atom utilization. Owing to the high precursor concentrations of Cu and Ni, the reduction kinetics were accelerated, leading to the preferential formation of Cu nanocrystalline seeds with a five‐fold twin structure [32]. These seeds subsequently served as sites for the reduction and epitaxial growth of other metal precursors introduced at lower concentrations. As a result, multi‐component nanoparticles were formed. Subsequent acid etching converted these nanoparticles into Pt‐based HENCs, which exhibit a highly defective structure, an enlarged surface area, and significant lattice strain.

The porous cage‐like morphology of PtPdNiCuMnAu HENCs is evident in the high‐angle annular dark‐field scanning transmission electron microscopy (HAADF‐STEM) images (Figure 1b,e). Further structural details are revealed by the high‐resolution images, which show distinct lattice fringes with ca. 0.216 nm (Figure 1c), corresponding to the (111) planes of the face‐centered cubic (*fcc*) phase. The *Z*‐contrast atomic‐resolution image (Figure 1d), where intensity is proportional to atomic numbers, reveals a random distribution of bright and dark atoms. This observation provides direct evidence for the synthesis of the Pt‐based high‐entropy alloys. PtPdNiCuMnAu HENCs form via the epitaxial growth step, preserving the twin boundary present in the nanocrystalline seeds. Furthermore, atomic defects are evident, manifested as disordered atomic arrangements and atomic vacancies that were likely introduced or amplified by the acid etching treatment. Elemental mapping (Figure 1f_1_–f_6_) confirms distinct growth mechanisms derived from the spatial distribution: the predominant edge distribution of Pt, Pd, and Cu signals epitaxial growth of Pt and Pd on the Cu seeds; in contrast, corner‐enriched Ni indicates its preferential deposition at high‐energy multiple‐twin sites. Meanwhile, the uniform distribution of Mn and Au suggests their random deposition on the seed surface.

TEM and STEM‐HAADF images show that PtPdNiCu NCs exhibit a similar cage‐like morphology (Figure 1g,h). The atomic‐resolution image further resolves the structure, revealing a lattice spacing of approximately 0.211 nm corresponding to the (111) facets of the *fcc* phase, alongside a random arrangement of atoms with varying Z‐contrast. PtPdNiCu NCs contained significantly fewer atomic vacancies than PtPdNiCuMnAu HENCs, owing to the absence of the highly reactive Mn. The analogous spatial distribution of the constituent elements is collectively confirmed by the corresponding elemental mappings (Figure 1l_1_–l_4_) and the EDS line scan profiles (Figure 1k) across the edge of PtPdNiCu NCs. To quantify the composition of each element, inductively coupled plasma optical emission spectrometry (ICP‐OES) was carried out. As shown in Figure 1m, the atomic percentages for PtPdNiCu NCs are determined to be 29.3% (Pt), 9.6% (Pd), 20.0% (Ni), and 41.1% (Cu). The corresponding values for PtPdNiCuMnAu HENCs are 23.0% (Pt), 12.6% (Pd), 15.6% (Ni), 44.2% (Cu), 3.3% (Mn) and 1.3% (Au). The detailed crystal structure was determined by X‐ray diffraction (XRD) analysis. As shown in Figure 1n and S2, both materials adopt the *fcc* crystal lattice system. The measured diffraction peaks of PtPdNiCu NCs lie between those of pure *fcc* Pt, Pd, Ni, and Cu phases. Specifically, the (111) peak shows a positive shift compared to Pt and Pd, and a negative shift compared to Ni and Cu. These features indicate the formation of a multi‐component solid solution. The measured (111) peak of PtPdNiCuMnAu HENCs lies between those of pure metal phases, showing a positive shift compared to Pt, Pd, and Au, as well as a negative shift compared to Ni, Cu, and Mn. Furthermore, relative to PtPdNiCu NCs, the (111) peak of PtPdNiCuMnAu HENCs exhibits noticeable broadening and a shift toward lower angles. This provides direct evidence for the associated lattice distortion and the enhanced atomic‐scale disorder. To quantitatively assess the structural lattice distortion and defect abundance in PtPdNiCuMnAu HENCs, the lattice micro‐strain (ε) was estimated from the XRD patterns using the Williamson‐Hall method applied to the (111) and (200) peaks (Table S2). The ε value was calculated to be 0.43% for PtPdNiCuMnAu HENCs, which is approximately 2.1 times higher than that of PtPdNiCu NCs (0.20%). This increased strain directly evidences the lattice distortion induced by the incorporation of Mn and Au, consistent with the observed (111) peak broadening and low‐angle shift. Notably, the XRD peak broadening after acid etching arises from the partial and preferential leaching of active transition metals, which induces local lattice strain and atomic defects, rather than poor crystallinity.

This defect‐rich architecture is conducive to exposing more atoms, such as Pt, to participate in catalytic reactions like ORR [24]. To probe the surface Pt sites, CO‐temperature‐programmed desorption (CO‐TPD) measurements were employed, leveraging the strong affinity of CO molecules for Pt atoms [33]. The nearly identical CO desorption temperatures (Figure 1o) for PtPdNiCuMnAu HENCs (401.0°C) and PtPdNiCu NCs (402.5°C) suggest a consistent linear adsorption configuration on Pt atoms [34]. To semi‐quantitatively evaluate the surface defect density, CO‐TPD curves were integrated after baseline subtraction to quantify the total CO desorption amount. Combined with the total Pt atom content determined by ICP‐OES and the sample amount, the Pt dispersion was calculated under the assumption of 1:1 CO/Pt adsorption stoichiometry and linear adsorption geometry (justified by the nearly identical CO desorption temperatures). Since defect sites such as atomic steps, kinks, and vacancies provide additional CO adsorption sites on Pt atoms, the Pt dispersion serves as a reliable indicator of the surface defect density. The Pt dispersion was significantly higher in PtPdNiCuMnAu HENCs (41.64%) than in PtPdNiCu NCs (33.94%). This 7.7% increase in Pt dispersion directly evidences the presence of additional surface defect sites arising from the synergistic effect of Mn and Au combined with acid etching, which is in full agreement with the atomic‐resolution HAADF‐STEM observations. The incorporation of Mn and Au has influenced the atomic arrangement in the polyhedra, assisted with acid etching treatment, therefore facilitating the exposure of a greater number of active Pt sites. This feature is conducive to providing more adsorption sites for oxygen molecules, ultimately improving the overall ORR efficiency.

**FIGURE 2:**
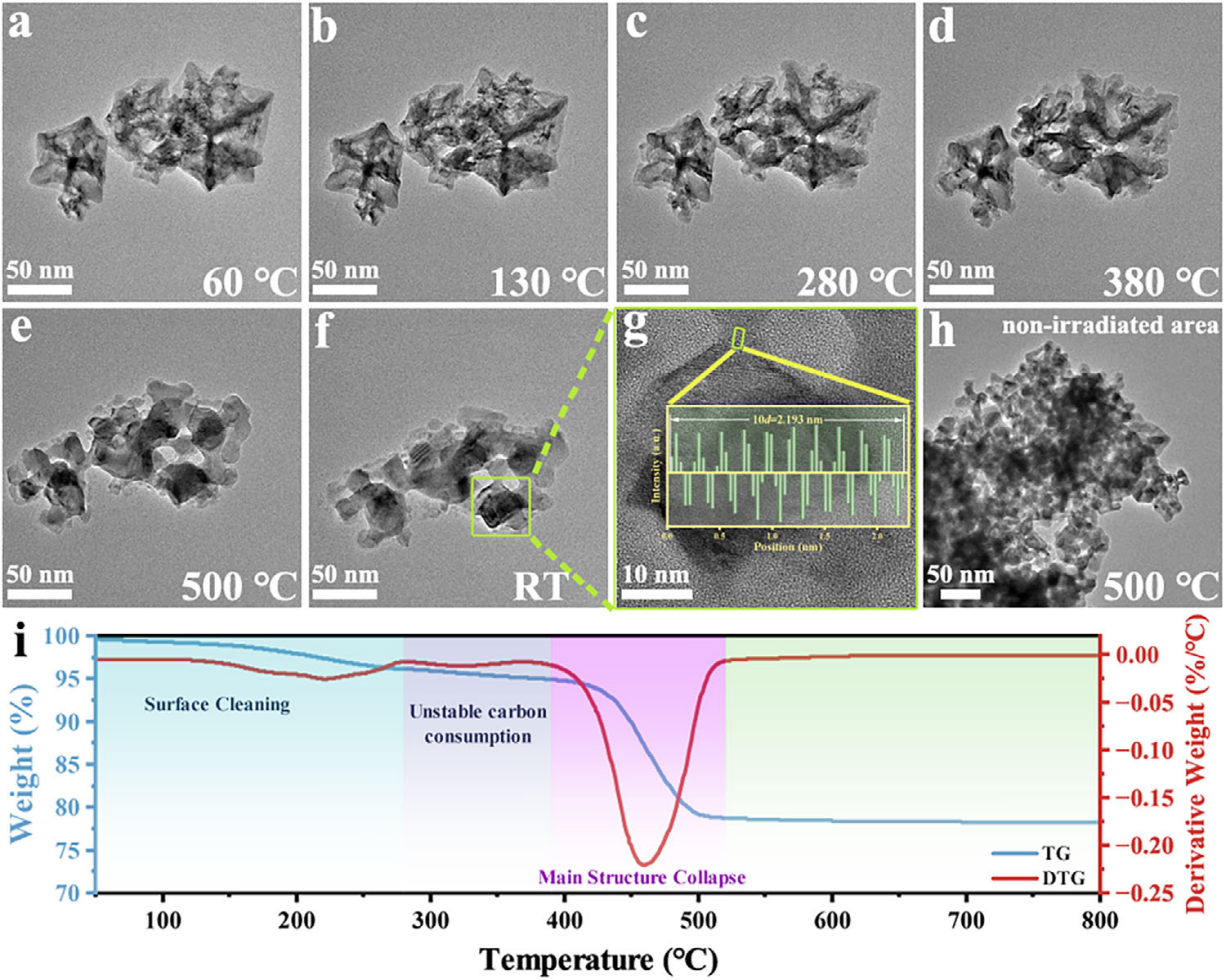
In situ heating TEM analysis and thermogravimetric result of PtPdNiCuMnAu HENCs: (a) 60°C, (b) 130°C, (c) 280°C, (d) 380°C, (e) 500°C, (f) area in (e) after cooling to room temperature, (g) magnified view, (h) 500°C in the non‐irradiated area, (i) TGA curve of PtPdNiCuMnAu HENCs/C.

Furthermore, to assess the applicability of PtPdNiCuMnAu HENCs, their thermal stability was evaluated. Excellent thermal stability, as a descriptor of strong atomic bonding and structural cohesion, is a cogent indicator of the potential for long‐term electrochemical stability of catalysts [35]. The thermal stability of the PtPdNiCuMnAu HENCs was systematically investigated: in situ heating TEM from 60°C to 500°C (Figures S3–S5) tracked the structural evolution, while thermogravimetric analysis (TGA) of the carbon‐supported sample (PtPdNiCuMnAu HENCs/C, Figure 2i) quantified the macroscopic weight loss. The cage‐like architecture remained intact up to 380°C (Figure 2a–d). The derivative thermogravimetry (DTG) curve shows the weight loss rate reached its first peak at around 280°C, with a total mass loss of 4% attributed to the evaporation of adsorbed water and decomposition of residual organics (Figure 2i). When further heated to 380°C, the mass loss further increased by 1%, likely due to the oxidation of amorphous carbon. The main structural collapse, accompanied by 16% mass loss, occurred between 380°C and 500°C (Figure 2e). It is noteworthy that electron beam irradiation during in situ TEM accelerates the sintering process. However, a small number of cage‐like structures can still be observed in non‐irradiated areas (Figure 2h). After cooling to room temperature, the high‐resolution image (Figure 2f,g) reveals a lattice spacing of ∼0.219 nm for the *fcc* (111) facets, slightly expanded from the initial 0.216 nm, indicating minor lattice relaxation. These results collectively demonstrate the excellent thermal stability of the PtPdNiCuMnAu HENCs, underscoring their potential for durable electrochemical applications.

**FIGURE 3:**
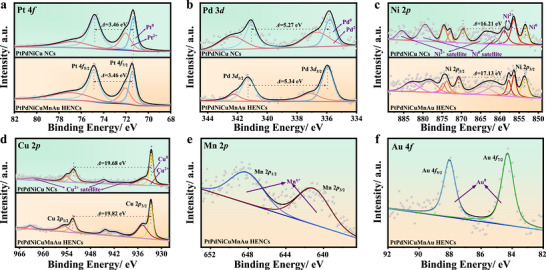
High‐resolution XPS spectra of PtPdNiCuMnAu HENCs and PtPdNiCu NCs: (a) Pt 4*f*, (b) Pd 3*d*, (c) Ni 2*p*, (d) Cu 2*p*, (e) Mn 2*p* and (f) Au 4*f*.

A key design principle for high‐performance ORR electrocatalysts is to achieve favorable oxygen binding characteristics, which involves maximizing O_2_ adsorption sites and moderating the binding strength of oxygen‐containing intermediates [36]. The surface electronic structure, a primary descriptor for the adsorption energy, was investigated via X‐ray photoelectron spectroscopy (XPS) to establish the structure‐activity relationship. The XPS survey spectra (Figure S6) reveal the presence of Pt, Pd, Ni, Cu, Mn and Au components. First of all, in the Pt 4*f* spectra, both catalysts exhibit doublets of Pt 4*f*_5/2_ and Pt 4*f*_7/2_, which can be deconvoluted into metallic Pt^0^ and oxidized Pt^2+^ species (Figure 3a). The presence of Pt^2+^ likely originates from oxidation in the air. Notably, distinct Ni^2+^ and Mn^x+^ peaks are observed (Figure 3c,e), indicating that the more oxidizable Ni and Mn preferentially undergo oxidation, thereby reducing the oxidation degree of Pt. Furthermore, the unsaturated coordination Pt atomic sites can elevate the binding energy of core‐level electrons, resulting in a Pt^2+^ like feature [37]. These sites are expected to not only facilitate the initial capture and activation of O_2_ molecules but also optimize the binding energies of key oxygen species [38]. This synergistic effect would lower the thermodynamic barrier of the rate‐determining step and promote product desorption, thereby enhancing the catalytic performance. The absence of manganese oxide diffraction peaks in the XRD pattern, combined with the ICP‐OES result, suggests that Mn atoms are highly dispersed and coordinated within the alloy.

The spin‐orbit splitting interval in the metallic state (*Δ*_M_) serves as another key indicator of the electronic structure [39, 40]. An increased *Δ*_M_ value occurs when a metal atom bonds with a more electronegative element. Consistent with this, Pd 3*d*, Ni 2*p*, and Cu 2*p* core levels in PtPdNiCuMnAu HENCs all shift to higher binding energies alongside increased *Δ*_M_ values, compared to PtPdNiCu NCs (Figure 3b–d). The presence of metallic Au in PtPdNiCuMnAu HENCs is confirmed by XPS (Figure 3f). Notably, the observed positive shift of the Au 4*f*_7/2_ peak (84.3 eV *vs*. standard 84.0 eV) is indicative of unsaturated coordination environments around Au atoms, leading to an electron‐deficient state. Compared to commercial Pt/C, Pt 4*f* peaks of both PtPdNiCuMnAu HENCs and PtPdNiCu NCs shift to lower binding energies (Figure S7). Although oxidized and unsaturated Pt sites represent localized electron‐deficient states, the overall electronic structure of Pt in alloys is modulated to be electron‐rich. This is driven by electron transfer from neighboring elements via the coordination effect (Table S3). The net result is a downshift of the Pt *d*‐band center, which optimizes the binding strength with reaction intermediates and improves the ORR performance.

**FIGURE 4:**
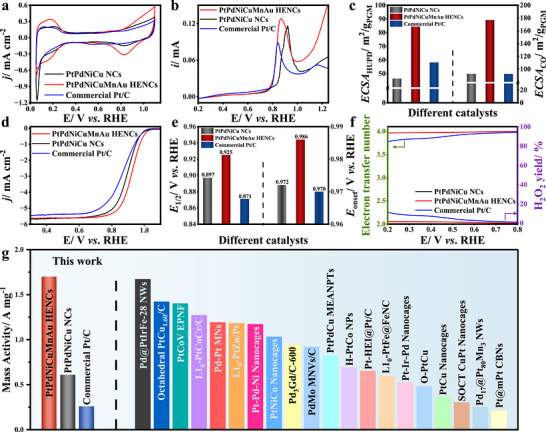
The comparison of electrochemical performance of PtPdNiCuMnAu HENCs, PtPdNiCu NCs and commercial Pt/C: (a) CV curves; (b) CO‐ stripping curves; (c) *ECSA*s determined by the HUPD and CO‐stripping methods; (d) LSV curves, (e) the half‐wave potential (*E*_1/2_) and the onset potential (*E*_onset_); (f) electron transfer number and H_2_O_2_ yield; (g) catalytic activity, including comparison with recently reported high‐entropy nanomaterials and noble‐metal nanocages.

The electrochemical ORR performance of PtPdNiCuMnAu HENCs and PtPdNiCu NCs was assessed in alkaline conditions using the rotating disk electrodes. To accurately determine the electrochemically active surface area (*ECSA*), cyclic voltammetry (CV) curves were first collected in N_2_‐saturated 0.5 m H_2_SO_4_ solution (Figure 4a) [41]. Based on the hydrogen adsorption/desorption regions, *ECSA*s of PtPdNiCuMnAu HENCs, PtPdNiCu NCs, and commercial Pt/C are calculated to be 84.25, 46.37, and 58.16 m^2^/g_PGM_, respectively (Figure 4c). It should be noted that hydrogen underpotential deposition‐derived *ECSA* values may be influenced by hydrogen diffusion into Pd lattices, leading to overestimation. Therefore, CO‐stripping voltammetry was employed as a complementary method to better reflect the active surface area and fine surface structure [42, 43]. As shown in Figure 4b,c, PtPdNiCuMnAu HENCs exhibit the highest *ECSA*_CO_ (176.56 m^2^/g_PGM_), together with a broad and asymmetrical CO oxidation peak and the lowest onset potential (*E*_onset_), collectively indicating enhanced intrinsic activity.

ORR polarization curves were obtained in O_2_‐saturated 0.1 m KOH solution at 1600 rpm with a scan rate of 20 mV/s (Figure 4d). PtPdNiCuMnAu HENCs exhibit the most positive *E*_onset_ (0.986 V *vs*. RHE) and half‐wave potential (*E*_1/2_, 0.925 V *vs*. RHE), outperforming both PtPdNiCu NCs (0.972 V *vs*. RHE, 0.897 V *vs*. RHE) and commercial Pt/C (0.970 V *vs*. RHE, 0.871 V *vs*. RHE) (Figure 4e). In terms of mass activity (MA) at 0.9 V *vs*. RHE, PtPdNiCuMnAu HENCs reach 1.70 A/mg_Pt_ (1.32 A/mg_PGM_), which is 7.39 times higher than that of commercial Pt/C (0.23 A/mg_Pt_) and also exceeds PtPdNiCu NCs (0.61 A/mg_Pt_, 0.51 A/mg_PGM_). This outstanding ORR performance positions PtPdNiCuMnAu HENCs among the top‐tier reported high‐entropy nanomaterials and cage‐shaped noble‐metal nanocatalysts (Figure 4g and Table S4). To probe the ORR pathway selectivity, the H_2_O_2_ yield was monitored during the reaction (Figure 4f). Compared to commercial Pt/C, both PtPdNiCuMnAu HENCs and PtPdNiCu NCs exhibit lower H_2_O_2_ yield and electron transfer numbers closer to 4.0 over the potential range of 0.2–0.8 V *vs*. RHE. These results confirm that the ORR process on PtPdNiCuMnAu HENCs and PtPdNiCu NCs primarily follows a highly selective four‐electron pathway, which directly reduces O_2_ to H_2_O rather than generating H_2_O_2_ via the two‐electron route, thereby ensuring higher energy conversion efficiency.

Similarly, the electrochemical ORR performance of PtPdNiCuMnAu HENCs and PtPdNiCu NCs was assessed in acid conditions using the rotating disk electrodes. As shown in Figure S8a, the hydrogen absorption/desorption area of PtPdNiCuMnAu HENCs is much larger than that of PtPdNiCu NCs and commercial Pt/C, indicating more exposed active sites on the HENCs surface. Peaks on CV curves corresponding to crystal facets are identified: (110) facets at ∼ 0.10 V *vs*. RHE, (111) facets at ∼ 0.18 V, and (100) facets at ∼0.23 V. Commercial Pt/C shows broad peaks for (110) and (100) facets, while both PtPdNiCuMnAu HENCs and PtPdNiCu NCs exhibit (110) and (111) facets. Notably, PtPdNiCuMnAu HENCs possess a much stronger (111) facet peak than PtPdNiCu NCs. As illustrated in Figure S8d, PtPdNiCuMnAu HENCs achieve the *ECSA*_HUPD_ of 92.1 m^2^/g_PGM_, higher than PtPdNiCu NCs (72.2 m^2^/g_PGM_) and commercial Pt/C (39.7 m^2^/g_PGM_). In the high potential region (0.7–1.1 V *vs*. RHE), the reduction peak of platinum/palladium oxide for PtPdNiCuMnAu HENCs appears at 0.816 V, which is negatively shifted relative to that of PtPdNiCu NCs. The defect sites on the surface of PtPdNiCuMnAu HENCs (twin boundary and atomic defects) possess slightly stronger oxygen binding ability, but are conducive to reduce the reaction activation energy, further promoting the ORR activity. Moreover, the incorporation of Mn and Au modulates the electronic structure of Pt sites and the following reaction pathway, invalidating the classic relationship between the reduction peak potential and ORR activity. In the O_2_‐saturated environment, the *E*_1/2_ of PtPdNiCuMnAu HENCs (0.905 V *vs*. RHE) is 99 mV more positive than that of commercial Pt/C (0.806 V *vs*. RHE) (Figure S8b,d). In the Tafel curves, the *j*_k_ at 0.9 V *vs*. RHE of PtPdNiCuMnAu HENCs is much more positive than that of commercial Pt/C and PtPdNiCu NCs, as well as the Tafel slope of PtPdNiCuMnAu HENCs is 67.5 mV/dec, smaller than 76.4 mV/dec of commercial Pt/C and 70.5 mV/dec of PtPdNiCu NCs (Figure S8c). The MA and SA of PtPdNiCuMnAu HENCs (0.985 A/mg_PGM_ and 1.07 mA/cm^2^) are 10.9 and 4.86 times higher than those of commercial Pt/C (0.09 A/mg_PGM_ and 0.22 mA/cm^2^), respectively. Turnover frequency (TOF) for PtPdNiCuMnAu HENCs and commercial Pt/C was further calculated. Commercial Pt/C has the lowest TOF value, while the TOF values of PtPdNiCuMnAu HENCs and PtPdNiCu NCs increased by 10.0 and 5.27 times, respectively (Figure S8d). The maximum TOF value of PtPdNiCuMnAu HENCs indicates the best intrinsic activity.

Due to the lower molar content of Mn and Au, the variation in the content of Pt, Pd, Ni, and Cu plays a more critical role in modulating the catalytic performance. Therefore, PtPdNiCu NCs with various elemental compositions were fabricated by tuning the acid etching conditions (Table S5) and utilized to evaluate the ORR performance. Figure S9 displays the correlation between their composition and electrochemical performance. With increasing configurational entropy, the *ECSA*_HUPD_ exhibits a clear monotonic increasing trend (from 38.9 to 72.2 m^2^/g_PGM_, Figure S9a,c). Similarly, the mass activity, intrinsic specific activity, and turnover frequency all increase monotonically as the configurational entropy rises. Specifically, the mass activity increases from 0.391 to 0.773 A/mg_Pt_, the specific activity from 0.747 to 0.902 mA/cm^2^, and the TOF from 0.98 to 1.16 s^−1^. These consistent monotonic trends indicate that increasing the configurational entropy systematically enhances both the *ECSA*_HUPD_ and the ORR kinetics.

To further elucidate the synergistic effects among constituent elements on the ORR performance, PtNiCu and PdNiCu catalysts were synthesized under the same conditions as PtPdNiCu NCs with the addition of only one precious metal precursor. As illustrated in Figures S10 and S11, both of them exhibit the well‐defined cage‐like architecture and homogeneous elemental distribution. Electrochemical characterization reveals that the hydrogen adsorption/desorption region of PtPdNiCu NCs is more pronounced than those of PtNiCu NCs and PdNiCu NCs (Figure S12a). Furthermore, the CO oxidation peak of PtPdNiCu NCs is located between those of the two reference catalysts (Figure S12b), suggesting a moderate electronic structure by the coexistence of Pt and Pd. The configurational entropy of PtPdNiCu NCs, calculated based on the elemental composition (Table S6), reaches 1.27*R*, significantly higher than those of PtNiCu NCs (0.70*R*) and PdNiCu NCs (0.88*R*). This noticeable increase in configurational entropy confirms a more randomized atomic arrangement, which favors the formation of a greater number of active sites. Benefiting from both the entropy effect and the electronic synergy, the PtPdNiCu NCs exhibit more positive *E*_o__n__s__e__t_ and *E*_1/2_ relative to the ternary counterparts (Figure S12c, Table S7).

In PtPdNiCuAu NCs, the Pt content significantly decreases to 12.3 at% compared to that in PtPdNiCu NCs. On the one hand, the high reduction potential of Au^3^^+^/Au (1.50 V *vs*. SHE) promotes preferential reduction and surface segregation of Au, forming a physical barrier that hinders Pt deposition. On the other hand, discontinuous Au distribution creates micro‐galvanic cells during acid etching, accelerating the dissolution of active metals and causing Pt loss. In contrast, PtPdNiCuMnAu HENCs exhibit a much higher Pt content of 26.8–28.9 at%, highlighting the key role of Mn in moderating surface segregation and dealloying. First, the low reduction potential of Mn^2^^+^/Mn (‐1.18 V *vs*. SHE) inhibits Au^3^^+^ reduction via complexation or local pH modulation, promoting uniform co‐deposition of Pt. Second, Mn and Au form stable intermetallic compounds (e.g., MnAu_2_ with formation energy ∼‐0.111 eV/atom) [44], which suppress Au segregation and anchor Au inside the alloy, exposing more Pt sites. Moreover, the configurational entropy of PtPdNiCuMnAu HENCs reaches 1.27‐1.44*R*, significantly higher than that of PtPdNiCuAu NCs (1.03*R*). This entropy‐driven homogenization promotes a uniform solid solution and suppresses element segregation. Additionally, lattice distortion and atomic defects increase the vacancy formation energy of Au, inhibiting its migration. The compressive strain induced by Mn enhances corrosion resistance, reduces non‐precious metal dissolution, and further stabilizes the skeleton.

The incorporation of Au atoms serves to increase the vacancy formation energy [9], which stabilizes the catalyst structure and ultimately improves the catalytic properties. Concurrently, Au is also expected to modulate the local electronic structure of PtPdNiCuMnAu HENCs, owing to its high electronegativity relative to the other constituent metals. To optimize the trade‐off between these structural and electronic effects, this study systematically tailored the Au content and evaluated its influence on the electrochemical performance. As evidenced in Figure S13a,b, the population of active sites is inversely correlated with the Au amount. Consistently, samples with excess Au exhibit a notable decrease in *E*_1/2_ (Figure S13c). Further structural characterization reveals that such Au‐rich samples possess expanded lattice spacing and distinct Au‐rich regions compared to the optimal catalyst (Figures S14 and S15). These findings collectively indicate that excessive Au tends to segregate to the surface, where it blocks active sites and consequently degrades the ORR activity.

In parallel to the surface‐related effects of Au, Mn incorporation was investigated for its role in engineering the bulk properties of Pt‐based HENCs. The distinct ionic radius and coordination preference of Mn introduce significant lattice strain and coordination defects, thereby creating a unique class of active sites. To elucidate this effect, this study compared the electrochemical performance of PtPdNiCuMnAu HENCs synthesized with varying molar amounts of the Mn^3+^ precursor. The elemental composition of each catalyst is summarized in Table S6. As shown in Figure S16a, the hydrogen adsorption/desorption regions of Mn‐incorporated HENCs are substantially enlarged compared to the Mn‐free PtPdNiCuAu benchmark, unequivocally confirming the role of Mn in generating additional active sites. Since the *ECSA*_HUPD_ is highly sensitive to the interfacial electronic effect and surface coverage, this notable enhancement is primarily attributed to the electronic effects modulated by Mn incorporation. This analysis is further supported by the XRD patterns (Figure S17), which exhibit the typical *fcc* phase diffraction peaks without detectable metal oxide phases, indicating that the alloy structural integrity is maintained and that the *ECSA* changes are not due to segregated oxide species. In contrast, the adsorption and subsequent oxidation of CO molecules occur preferentially on continuous Pt atoms, rendering *ECSA*_CO_ more sensitive to the geometric arrangement and dispersion of Pt sites (Figure S16b). The synergistic effects of the enhanced *ECSA* and the optimized electronic environment culminate in superior ORR activity. As shown in Figure S16c and Table S7, ORR polarization curves demonstrate that the introduction of Mn induces a positive shift of 7 to 43 mV in *E*_1/2_, underscoring the significant promotional effect of Mn doping. Further analysis reveals a strong positive correlation between the ratio of *ECSA*_HUPD_/*ECSA*_CO_ and the Pt loading, which in turn exhibits a volcanic relationship with the corresponding MA at 0.9 V *vs*. RHE (Figure S16d).

**FIGURE 5:**
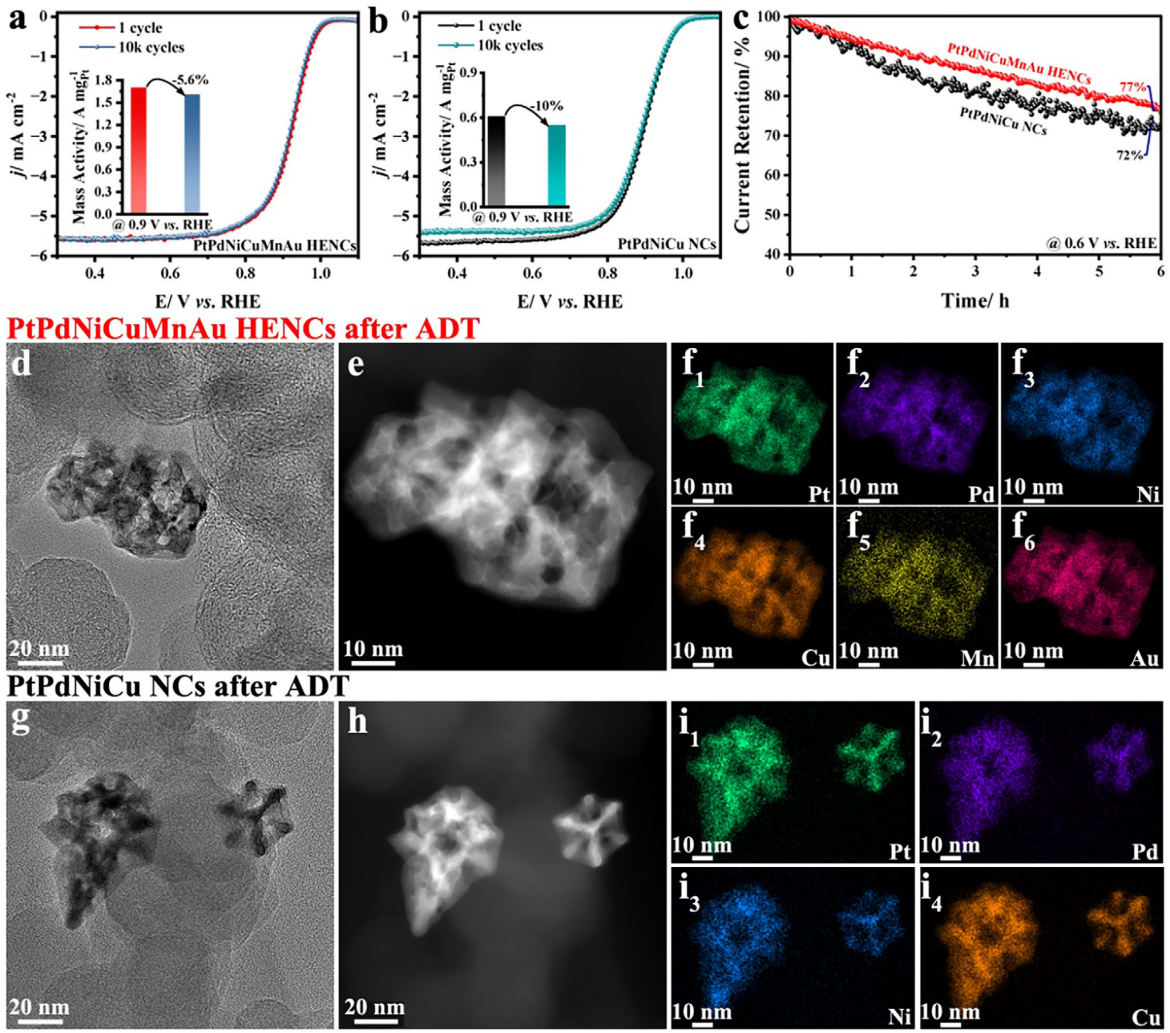
Analysis of electrochemical performance for PtPdNiCuMnAu HENCs and PtPdNiCu NCs: (a, b) comparison of LSV curves before and after accelerated durability tests, the inset shows the evolution of mass activity at 0.9 V *vs*. RHE; (c) chronoamperometric tests measured at 0.6 V *vs*. RHE; (d) TEM image, (e) STEM‐HAADF image and (f_1_‐f_6_) corresponding elemental mappings of PtPdNiCuMnAu HENCs after ADT cycles; (g) TEM image, (h) STEM‐HAADF image and (i_1_‐i_4_) corresponding elemental mappings of PtPdNiCu NCs after ADT cycles.

Complementing exceptional performance, long‐term durability is another crucial criterion for evaluating ORR catalysts. The accelerated durability test (ADT) was conducted by cycling the potential between 0.6–1.1 V *vs*. RHE in N_2_‐saturated 0.1 m KOH with a scan rate of 100 mV/s. After 10 000 cycles, PtPdNiCuMnAu HENCs show only a 4 mV negative shift in *E*_1/2_ and a 5.6% loss in MA (Figure 5a). In contrast, PtPdNiCu NCs exhibit a more pronounced degradation, with a 9 mV decrease in *E*_1/2_ and a 10% drop in MA (Figure 5b). Commercial Pt/C suffered from severe performance decay, which is collectively demonstrated by a 33 mV decrease in *E*_1/2_ and a 71.7% reduction in MA (Figure S18a). This interior stability is likely attributable to the dissolution of active components, exacerbated by its lower configurational entropy relative to the HENCs. Chronoamperometric measurements at 0.6 V *vs*. RHE further confirm the enhanced durability of PtPdNiCuMnAu HENCs, which retain 77% of the initial current after 6 h of continuous operation, outperforming PtPdNiCu NCs (Figure 5c). Under the same conditions, commercial Pt/C suffers a rapid performance decay, retaining only 36% of its activity after merely 2 h (Figure S18b).

Post‐ADT structural characterization reveals that PtPdNiCuMnAu HENCs maintain their original cage‐like morphology (Figure 5d,e) with no significant elemental segregation, as evidenced by homogeneous elemental distribution (Figure 5f_1_–f_6_ and Figure S19). This structural integrity from various random areas, combined with a retained high configurational entropy (1.31*R*, Table S5), directly demonstrates the entropy‐stabilization effect in the PtPdNiCuMnAu solid solutions. Although PtPdNiCu NCs also preserve their overall geometric structure (Figure 5g,h), local elemental segregation is observed in corresponding EDS mappings, providing a plausible explanation for their activity decay (Figure S19).

Complementary ICP‐OES/MS analysis (Table S9) quantifies the compositional evolution before and after ADT, explicitly identifying the most vulnerable species. In the baseline PtPdNiCu system, the total metal leaching amount reaches 20.3%. Among all components, Cu exhibits the highest leaching ratio of 26.4% and a relative atomic ratio decrease of 10.5% *vs*. Pt after ADT (Figure S21a). These results collectively confirm Cu as the primary trigger for catalyst deactivation. As shown in Figure S21b, the sole addition of Mn unexpectedly exacerbates Ni and Cu leaching (44.4% and 47.0%, respectively), attributed to the chemical activity of Mn and surface reconstruction under potential cycling. In contrast, PtPdNiCuMnAu HENCs exhibit substantially suppressed dissolution: Ni and Cu leaching ratios drop to 9.05% and 10.5%, respectively, and the relative atomic ratio of Cu decreases by only 8.85% after ADT (Figure S21c). These data demonstrate that the incorporation of Au not only preserves Mn during acid etching but also synergistically inhibits Cu oxidative dissolution, overcoming the catalytic bucket effect.

These results validate the lattice‐stabilizing effect of Au, and the Au pinning structure elevates the energy barrier for atomic migration and dissolution during acid etching and potential cycling. The XRD peak broadening induced by acid etching does not contradict the lattice stabilization provided by Au, because the peak broadening would be far more severe and even cause structural failure without Au. The exceptional stability of PtPdNiCuMnAu HENCs arises from the synergistic interplay of multiple strain effects. Alloying Pt with other transition metals generates macro‐strain and ligand effects, which initially enhance ORR activity. These benefits gradually fade as active metals dissolve or segregate during cycling. The exceptional stability of PtPdNiCuMnAu HENCs arises from the synergistic interplay of multiple strain effects. Alloying Pt with other transition metals generates macro‐strain and ligand effects that initially boost ORR activity, while these effects gradually weaken as active metals dissolve or segregate during cycling. The robust structural stabilization mainly derives from the surface micro‐strain induced by Au and Mn, which effectively suppresses the loss of active sites. The high‐entropy matrix plays a decisive role, and its retained configurational entropy (1.31*R* after ADT cycling) hinders elemental diffusion and segregation. Post‐ADT mapping results verify the formation of a uniformly distributed solid solution. The dual mechanism that combines surface micro‐strain and bulk entropy stabilization collectively endows the HENCs with outstanding operational durability, which is far superior to PtPdNiCu NCs and commercial Pt/C.

**FIGURE 6:**
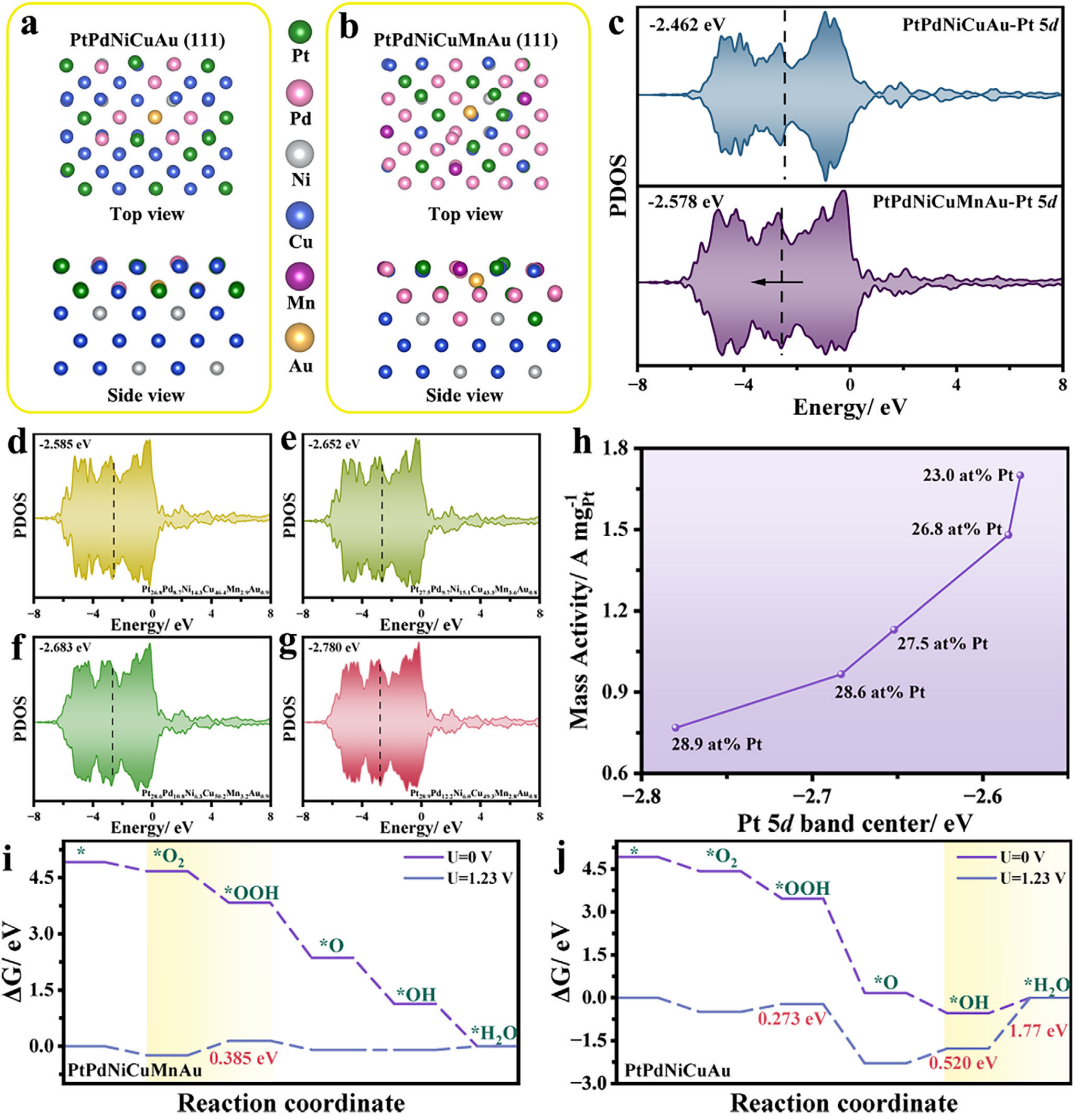
ORR mechanistic investigation on PtPdNiCuMnAu high‐entropy alloys: top and side views of optimized (111) surface models for (a) PtPdNiCuAu and (b) PtPdNiCuMnAu; (c) comparison of PDOS of Pt sites in PtPdNiCuAu and PtPdNiCuMnAu; (d‐g) composition‐dependent evolution of PDOS of Pt sites; (h) the relationship between the calculated Pt *d*‐band center and the experimental mass activity across the different compositions; Gibbs free energy diagram at 0 and 1.23 V of (i) PtPdNiCuMnAu and (j) PtPdNiCuAu, respectively.

DFT calculations were employed to elucidate the mechanism behind the enhanced ORR performance of PtPdNiCuMnAu high‐entropy alloys. Based on the experimental results, a series of structural models were constructed, including the (111) surfaces of PtPdNiCuMnAu with varying compositions, and a reference PtPdNiCuAu surface (Figure 6a,b and Figure S22). The projected density of states (PDOS) was analyzed to gain insights into the adsorption behavior at the surface Pt sites. As displayed in Figure 6c, the *d*‐band centers of Pt sites in PtPdNiCuMnAu and PtPdNiCuAu shift downward by 0.328 and 0.212 eV, respectively, compared to that of pure Pt (‐2.25 eV) [27, 45]. This shift can be attributed to the electron transfer within the complex multi‐element surface, which collectively modulates the electronic structure of Pt. The downward shift of *d*‐band centers is expected to weaken the binding strength between Pt sites and oxygen‐containing intermediates, thereby facilitating the ORR kinetics [46]. Furthermore, the Pt *d*‐band center of PtPdNiCuMnAu surfaces with different compositions is consistently lower than that of the Mn‐free PtPdNiCuAu benchmark (Figure 6d–g), underscoring the critical role of Mn incorporation in fine‐tuning the surface electronic structure. Notably, a linear correlation was observed between the experimental ORR activity of PtPdNiCuMnAu HENCs and the calculated Pt *d*‐band center across the different compositions (Figure 6h). This compelling relationship conclusively indicates that the ORR activity is primarily governed by the electronic structure of Pt sites, with Mn incorporation being a key compositional parameter for its systematic optimization.

Given that the ORR involves multiple electron transfer steps, the Gibbs free energies of the intermediate products are critical to elucidating its underlying mechanism. The results presented above collectively demonstrate that the ORR on Pt‐based alloys proceeds via the four‐electron pathway. As shown in Figure 6i, the energy paths of PtPdNiCuMnAu at U = 0 V show a spontaneous exothermic process. Furthermore, at an equilibrium potential of U = 1.23 V, the rate‐limiting step is the hydrogenation of ^*^O_2_ to form the ^*^OOH intermediate with a ΔG of 0.385 eV. For PtPdNiCuAu, the free energy rise in the protonation of ^*^OH is the largest (1.77 eV) and therefore is the decisive step. These results indicate that the incorporation of Mn not only reduces the d‐band center and optimizes the binding strength with oxygen intermediates, but also lowers the energy barrier of the protonation process, promoting reaction kinetics.

**FIGURE 7:**
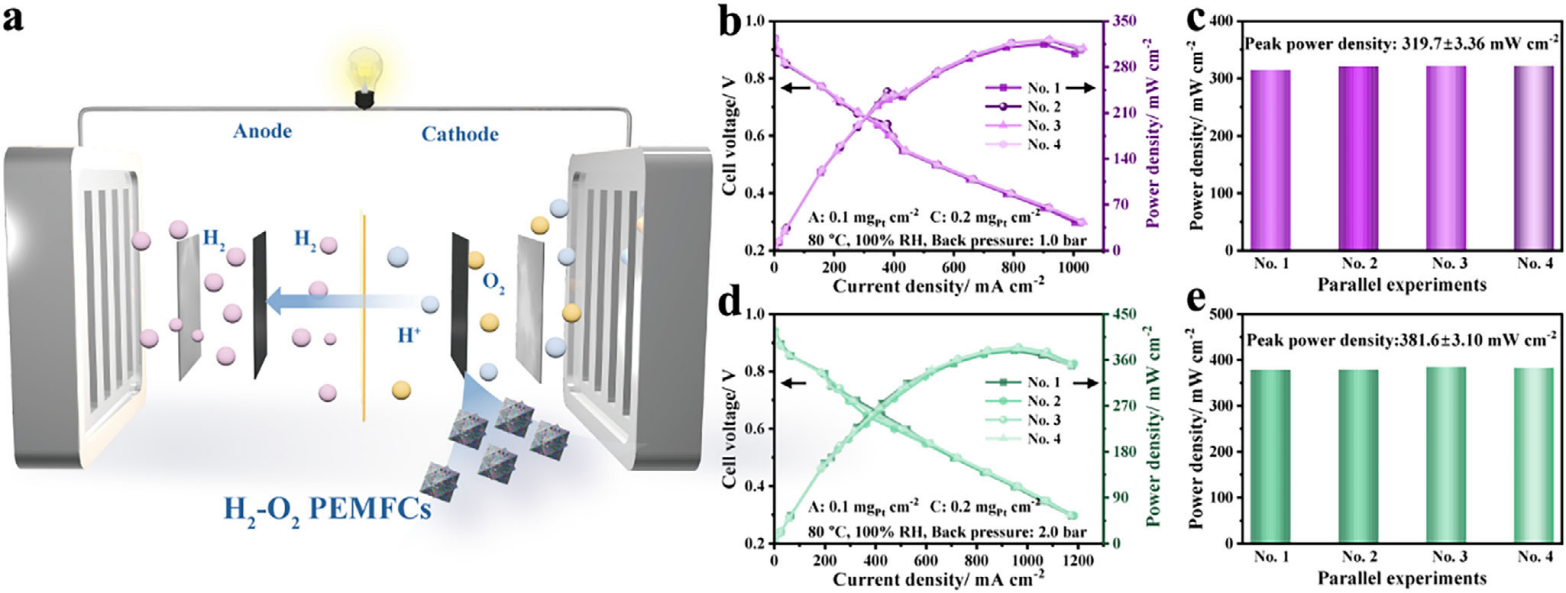
(a) The structure of H_2_‐O_2_ PEMFCs for PtPdNiCuMnAu HENCs as cathodes. (b) I‐V polarization and power density plots with 1.0 bar back pressure, (c) the histogram of the peak power density under 1.0 bar back pressure for parallel experiments. (d) I‐V polarization and power density plots with 2.0 bar back pressure, (e) the histogram of the peak power density under 2.0 bar back pressure for parallel experiments.

To assess the application potential of Pt‐based high‐entropy catalysts in PEMFCs, the membrane electrode assembly (MEA) was prepared with an anode loading of 0.1 mg_Pt_ cm^−2^ for 20% Pt/C and a cathode loading of 0.2 mg_Pt_ cm^−2^ for PtPdNiCuMnAu HENCs (Figure 7a). The proper back pressure facilitates gas diffusion kinetics and avoids polarization effects. On the other hand, it also refines the water management, balancing membrane resistance and water logging. To avoid single‐experiment errors, Figure 7b,d display *I–V* polarization and power density plots for four parallel experiments under different back pressures. In H_2_‐O_2_ conditions, PtPdNiCuMnAu HENCs achieve the peak power density of 319.7 ± 3.36 mW cm^−2^ under 1.0 bar back pressure (Figure 7c) and 381.6 ± 3.10 mW cm^−2^ under 2.0 bar back pressure (Figure 7e), respectively. For comparison, the MEA performance of PtPdNiCu NCs was measured under identical conditions, showing peak densities of 235 and 261 mW cm^−2^ under 1.0 and 2.0 bar, respectively (Figure S23). Despite comparable intrinsic activity in three‐electrode tests, PtPdNiCuMnAu HENCs and PtPdNiCu NCs diverge markedly under harsher MEA conditions (severe mass transfer, high local current density, and three‐phase interface fluctuations). The high configuration entropy combined with the Mn and Au atomic pinning effect in PtPdNiCuMnAu HENCs effectively suppresses transition metal dissolution, delivering substantially higher power densities. These results demonstrate that the high‐entropy strategy plays a key role in designing efficient and durable multicomponent alloys for real fuel cell environments.

## 3 Conclusion

In summary, we have successfully synthesized porous and cage‐like PtPdNiCuMnAu HENCs through a combined strategy of liquid‐phase reduction and acid etching. The catalyst exhibits distinct structural characteristics‐including a high density of atomic defects, lattice strain, and an enlarged surface area‐arising from the synergistic cocktail effect of high‐entropy alloys and the selective etching process. PtPdNiCuMnAu HENCs demonstrate outstanding electrocatalytic performance for ORR, achieving an *E*_1/2_ of 0.925 V *vs*. RHE and a superior mass activity of 1.70 A/mg_Pt_, which are 7.39 and 2.80 times higher than those of commercial Pt/C and PtPdNiCu NCs, respectively. Moreover, the catalyst exhibits excellent stability, with only a 4 mV negative shift in *E*_1/2_ and a 5.6% loss in mass activity after 10 000 cycles. The H_2_‐O_2_ PEMFC performance of PtPdNiCuMnAu HENCs rose to 381.6 ± 3.10 mW cm^−2^ at 2.0 bar back pressure, notably higher than 319.7 ± 3.36 mW cm^−2^ achieved at 1.0 bar. This further highlights its latent capacity for performance optimization under elevated pressure. Through multi‐scale characterization and DFT calculations, we have elucidated the underlying mechanisms for the enhanced ORR performance: (1) lattice distortion and atomic vacancies induced by acid etching expose more active sites and optimize the adsorption behavior of oxygenated intermediates; (2) the incorporation of Mn and Au downshifts the Pt *d*‐band center, weakening the binding strength of key intermediates and accelerating reaction kinetics; (3) the homogeneous multielement distribution within the high‐entropy solid solution structure effectively suppresses metal dissolution and segregation, thereby enhancing structural durability; (4) the increased vacancy formation energy contributed by Au and the pronounced lattice distortion induced by Mn collectively expand the number of active sites and boost intrinsic activity. This study not only presents a class of cage‐like high‐entropy catalysts with exceptional ORR performance, but also establishes a theoretical foundation and material platform for the rational design of high‐entropy electrocatalysts that simultaneously achieve high activity and stability, guided by multi‐scale structure‐performance correlations.

## Author Contributions

**Yiou Liu**: data curation. **Xiaowei Zhang**: formal analysis. **Xu Chen**: data curation. **Haoran Kang**: data curation. **Qian Liu**: investigation, writing – original draft, writing – review and editing, validation, methodology, visualization, software, resources, project administration. **Faming Gao**: conceptualization, writing – review and editing, funding acquisition, supervision.

## Conflicts of Interest

The authors declare no conflicts of interest.

## Supporting information




**Supporting File**: advs76501‐sup‐0001‐SuppMat.docx.

## Data Availability

The data that support the findings of this study are available from the corresponding author upon reasonable request.
