# Supplementary material for: Overcoming the Catalytic Bucket Effect in Pt‐based High‐Entropy Nanocages Through Interface Defect and Strain Engineering
Source: Adv Sci (Weinh). 2026 Jul 8;13(55):e76501. doi: 10.1002/advs.76501 (PMC13344506; doi:10.1002/advs.76501)
Supplement: Supplementary file 1 [file ADVS-13-e76501-s001.docx]

**Supporting Information**

**Overcoming the Catalytic Bucket Effect in Pt-based High-Entropy Nanocages through Interface Defect and Strain Engineering**

Qian Liu^1,^ *, Haoran Kang^1^, Yiou Liu^1^, Xiaowei Zhang^1^, Xu Chen^1^,, Faming Gao^1, 2,^ *

^1^Tianjin Key Laboratory of Multiplexed Identiﬁcation for Port Hazardous Chemicals, State Key Laboratory of Bio-based Fiber Materials, Tianjin University of Science & Technology, Tianjin 300222, P. R. China

^2^Hebei Key Laboratory of Applied Chemistry, Yanshan University, Qinhuangdao 066004, China

***E-mail addresses**

fmgao@tust.edu.cn (Faming Gao)

**This PDF file includes:**

1. Materials and reagents

2. Methods

3. Instruments

4. Electrochemical measurements

5. Figure S1-S23

6. Table S1-S9

**1. Materials and reagents**

Platinum acetylacetonate (Pt(acac)_2_, 97%), palladium acetylacetonate (Pd(acac)_2_, 99%), manganic acetylacetonate (Mn(acac)3, 97%), nickel acetylacetonate (Ni(acac)2, 95%), copper chloride dihydrate (CuCl_2_·2H_2_O, AR), gold chloride trihydrate (HAuCl_4_·3H_2_O, 99.5%), potassium hydroxide (KOH, 85%), cetyltrimethylammonium chloride (CTAC, 97%), oleylamine (OAm, C18 80%-90%), benzyl alcohol (98%) and Nafion (5 wt%) used in this work were purchased from Aladdin (Shanghai, China). Cyclohexane (AR), ethanol (AR), isopropanol (AR) were obtained from Tianjin Kemiou Chemical Reagent Co. Ltd. (Tianjin, China). Nitric acid (HNO_3_, GR) and sulfuric acid (H_2_SO_4_, GR) were obtained from China National Pharmaceutical Group Corporation. Wahaha Pure Water from Wahaha Group Co. Commercial Pt/C catalysts (20 wt%, Johnson Matthey). All reagents were used without further purification.

**2. Methods**

In the typical synthesis of PtPdNiCuMnAu HENCs, 8 mg Pt(acac)_2_, 1.7 mg Pd(acac)_2_, 5 mg Mn(acac)_3_, 10.8 mg Ni(acac)_2_, 11.8 mg CuCl_2_·2H_2_O, 19.2 mg CTAC and 6 mL OAm were mixed together in a 10 mL centrifuge tube and ultrasonicated for around 1h to form a homogeneous turquoise mixture solution A. Dissolve a certain amount of HAuCl_4_·3H_2_O in benzyl alcohol to form a solution B with a concentration of 40 mmol/L. Pipette 12.5 uL of solution B into solution A, and label it as the precursor solution. Then, 20 mL benzyl alcohol was added to a 100 mL three-necked flask and preheated at 185°C for 10 min with strongly magnetic stirring. After that, the precursor solution was dropped into the flask evenly at the rate of 3 mL per an hour and kept the temperature for another 15 min. After cooling to the room temperature, the products were collected by using centrifugation at 4000 rpm for 10 min with cyclohexane and ethanol, respectively. Products were redissolved in 15 mL ethanol with strongly magnetic stirring for 5min while adding 2mL HNO_3_ and 2mL HAc to etch inner Cu. The black products were separated using centrifugation at 4000 rpm and washed several times with ethanol.

In the typical synthesis of carbon supported catalysts, XC-72 carbon was dispersed in the anhydrous ethanol under ultrasonication for 6 h. Then, the mixture was dropwise added into the ethanol solution with dispersed PtPdNiCuMnAu HENCs under magnetic stirring for 12 h. The catalysts with carbon were collected by centrifugation at 9500 rpm for 15 min. Finally, PtPdNiCuMnAu HENCs/ C were centrifuged and washed with ethanol, and dried at 60 °C in an oven overnight.

The synthesis of PtPdNiCu NCs and PtPdNiCuMnAu HENCs with different composition is similar to that of PtPdNiCuMnAu HENCs, only the type and dosage of the precursors are altered (**Table S5**).

**3. Instruments**

The morphology and microstructure were examined by means of transmission electron microscopy (HITACHI HT7700 operated at 120 kV) and X-ray diffraction measurements (SmartLab, Rigaku equipped with a Cu Kα X-ray radiation source). High-angle annular dark-field scanning transmission electron microscopy (HAADF-STEM) and high-resolution TEM (HRTEM) were carried out on Spectra300. The fabrication and *in-situ* heating testing of PtPdNiCuMnAu HENCs were conducted utilizing a Zeptools PicoFemto® atmosphere heating single-tilt holder with an Spectra300 transmission electron microscope operating at 300 kV. XPS measurements were performed on ESCALAB 250Xi spectrometer (Thermo Fisher Scientific) using Al Kα radiation with 1486.68 eV. The weight ratios of elements were revealed by inductively coupled plasma atomic emission spectrometer (Agilent ICP-AES 725ES). The thermal properties were evaluated on an SDT-Q600 thermogravimetric analyzer (TA Instruments) from 30 to 800 °C with a heating rate of 20 °C/min under a N_2_ atmosphere.

**4. TPD measurement**

For the CO-temperature programmed desorption (CO-TPD) measurement, 70 mg catalyst was placed at the bottom of the U-shaped quartz tube and pretreated in the He flow at 200 °C for 1h before cooling down to room temperature. Subsequently, a mixture of 5% CO/He flowed through a catalyst bed at room temperature for 0.5 h at a flow rate of 50 mL/min. Then, He flow was used to remove the physisorbed CO for 40 min at a flow rate of 30 mL/min. Finally, the catalyst bed was heated from room temperature to 400 °C at 10 °C/min.

**5. Electrochemical measurements**

The electrochemical performance of catalysts was performed on a CHI760E instrument as well as a rotating risk electrode. The catalysts were dispersed into the mixture of isopropanol, water and Nafion (v:v:v=1:1:0.02) to form a catalyst ink with the concentration of 2 mg mL^-1^. Before electrochemical tests, a glass carbon electrode (GCE) was polished with alumina of 0.5 μm and 0.05 μm in turn. In addition, 10 μL of catalyst ink was deposited onto the surface of GCE.

The cyclic voltammetry (CV) curves were recorded in the N_2_-purged 0.5 M H_2_SO_4_ solution in the potential range of 0.05-1.1 V *vs*. RHE at the scanning rate of 50 mV s^-1^. In this three-electrode system, the reference electrode was a Hg/Hg_2_SO_4_ electrode, and the counter electrode was a platinum plate.

The CO stripping measurement was first performed with a chronoamperometry test at 0.2 V *vs*. Hg/Hg_2_SO_4_ for 1200s in a CO-saturated 0.5 M H_2_SO_4_ solution for CO adsorption. Then, the working electrode was transferred to fresh 0.5 M H_2_SO_4_, and two cycles were recorded with a scan rate of 20 mV s^-1^ to determine the peak of CO stripping.

The oxygen reduction reaction measurement was performed at room temperature using RDE at a scan rate of 20 mV s^-1^ with 1600 rpm in the O_2_-purged 0.1 M KOH. In this three-electrode system, the reference electrode was a Hg/HgO electrode, and the counter electrode was a platinum plate. Accelerated durability tests were conducted by cycling the potential between 0.6-1.1 V *vs*. RHE at 500 mV s^-1^ for 30,000 cycles. The electron transfer number and H_2_O_2_ yield measurement were determined using a rotating ring-disk electrode (RRDE) in O_2_-purged 0.1 M KOH at room temperature. The measurements were conducted with a scan rate of 20 mV s^-1^ and a rotation speed of 1600 rpm, while the ring potential was held constant at 1.3 V *vs*. RHE. I-t curves were recorded at a constant potential of 0.6 V *vs*. RHE with 1600 rpm in the O_2_-purged 0.1 M KOH for a period of 6 hours.

**6. DFT setup**

DFT calculations were performed using the Vienna ab initio simulation package (VASP). The generalized gradient approximation (GGA) in the form of Perdew-Burke-Ernzerhof (PBE) was adopted as the exchange correlation functional. The energy cutoff of 450 eV and the k-point meshes of 1×1×1 were proposed to carry out geometry optimization and electronic structure calculation. During the geometry optimization, the entire system is considered to have successfully converged until the convergence thresholds of maximum force and energy were smaller than 0.05 eV/ A and 2.0×10-5 eV/atom, respectively. The vacuum slab was set as 10Å to avoid interactions between neighboring structures.

**7. Membrane electrode assembly (MEA) preparation and measurements**

MEAs were fabricated with PtPdNiCuMnAu HENCs as the cathode catalyst (0.2 mg_Pt_ cm^-2^) and commercial 20 wt.% Pt/C as the anode catalyst (0.1 mg_Pt_ cm^-2^). Their PEMFC performance was then evaluated under H_2_-O_2_ conditions. The catalyst inks were prepared by mixing the catalyst with 5% Nafion dispersion, isopropanol and deionized water (v:v:v=24:125:51). MEAs were prepared by assembling in order the gasket, carbon paper (205 μm, Avcarb), anodic catalyst, PEM (25.4 μm, Suzhou Sinero), cathodic catalyst, carbon paper and gasket. The catalyst inks were sprayed onto a 3×3 cm^2^ proton exchange membrane at 70°C. The PEMFCs performance of H_2_-O_2_ (0.2/0.4 L/min) was measured using a fuel cell tester at 80°C, 100% relative humidity, and a back pressure of 1.0/2.0 bar. Before the polarization curves were recorded, the MEA was fully activated by holding at 0.6 V and 0.4 V for 10 min for 10 cycles to stabilize the potential and current density.

**5. Figures**

**Figure S1.** HAADF image and corresponding EDS mapping results of PtPdNiCuMnAu nanocrystals obtained from the drip strategy (**a-g**) and one-pot strategy (**h-n**).

**Figure S2.** XRD pattern of PtPdNiCuMnAu HENCs.

**Figure S3**. *In*-situ heating TEM analysis and thermogravimetric result of PtPdNiCuMnAu HENCs: (**a**) 60 ℃, (**b**) 70 ℃, (**c**) 80 ℃, (**d**) 90 ℃, (**e**) 100 ℃, (**f**) 110 ℃, (**g**) 120 ℃, (**h**) 130 ℃, (**i**) 140 ℃, (**j**) 150 ℃, (**k**) 160 ℃, (**l**) 170 ℃, (**m**) 180 ℃, (**n**) 190 ℃, (**o**) 200 ℃.

**Figure S4**. *In*-situ heating TEM analysis and thermogravimetric result of PtPdNiCuMnAu HENCs: (**a**) 210 ℃, (**b**) 220 ℃, (**c**) 230 ℃, (**d**) 240 ℃, (**e**) 250 ℃, (**f**) 260 ℃, (**g**) 270 ℃, (**h**) 280 ℃, (**i**) 290 ℃, (**j**) 300 ℃, (**k**) 310 ℃, (**l**) 320 ℃, (**m**) 330 ℃, (**n**) 340 ℃, (**o**) 350 ℃.

**Figure S5**. *In*-situ heating TEM analysis and thermogravimetric result of PtPdNiCuMnAu HENCs: (**a**) 360 ℃, (**b**) 370 ℃, (**c**) 380 ℃, (**d**) 390 ℃, (**e**) 400 ℃, (**f**) 410 ℃, (**g**) 420 ℃, (**h**) 430 ℃, (**i**) 440 ℃, (**j**) 450 ℃, (**k**) 460 ℃, (**l**) 470 ℃, (**m**) 480 ℃, (**n**) 490 ℃, (**o**) 500 ℃.

**Figure S6.** XPS survey spectra of (**a**) PtPdNiCuMnAu HENCs and (**b**) PtPdNiCu NCs.

**Figure S7.** (**a**)The comparison of high-resolution Pt 4*f* spectra of PtPdNiCuMnAu HENCs, PtPdNiCu NCs and commercial Pt/C; (**b**) high-resolution Pt 4*f* spectra of commercial Pt/C.

**Figure S8.** The ORR performance of PtPdNiCu NCs, PtPdNiCuMnAu HENCs and commercial Pt/C measured in 0.1 M HClO_4_ solution: **(a)** CV curves, **(b)** LSV curves, **(c)** Tafel plots and **(d)** the comprehensive performance comparison.

**Figure S9.** The electrochemical performance comparison of PtPdNiCu NCs with different atomic ratios in 0.1 M HClO_4_ solution: **(a)** CV curves, **(b)** LSV curves and **(c)** the summary of *ECSA*_HUPD_, ORR kinetic parameters and configurational entropy.

**Figure S10.** The characterization of PtNiCu NCs: (**a**, **b**) HAADF-STEM images, (**c_1_**-**c_3_**) corresponding elemental mappings, (**d**) elemental line-scan profiles along the yellow arrow in **b**.

**Figure S11.** The characterization of PdNiCu NCs: (**a)** TEM image, (**b**) HAADF-STEM image, (**c_1_**-**c_3_**) corresponding elemental mappings, (**d**) elemental line-scan profiles along the pink arrow in **b**.

**Figure S12**. The synergistic effect of multiple components on electrochemical performance: (a) CV curves; (b) CO stripping curves; (c) LSV curves.

**Figure S13**. The effect of the addition amount of Au^3+^ precursor on electrochemical performance: (a) CV curves; (b) CO stripping curves; (c) LSV curves.

**Figure S14**. The characterization of PtPdNiCuMnAu HENCs within 1.0 μmol Au^3+^ precursors: (**a**, **b**) low resolution HAADF-STEM images, (**c**) high resolution HAADF-STEM image, (**d_1_**-**d_6_**) corresponding elemental mapping.

**Figure S15**. The characterization of PtPdNiCuMnAu HENCs within 1.5 μmol Au^3+^ precursors: (**a**) low resolution HAADF-STEM image, (**b_1_**-**b_6_**) corresponding elemental mapping.

**Figure S16**. The electrochemical performance comparison of PtPdNiCuMnAu HENCs with different molar amount of Mn^3+^ precursor: (**a**) CV curves; (**b**) CO-stripping curves; (**c**) LSV curves; (**d**) the value of *ECSA*_HUPD_/*ECSA*_CO_ (left) and mass activity at 0.9 V vs. RHE (right) of different catalysts.

**Figure S17**. XRD patterns of PtPdNiCuMnAu HENCs with different molar amount of Mn^3+^ precursor.

**Figure S18**. (**a**) Comparison of LSV curves before and after accelerated durability tests, the inset shows the evolution of mass activity at 0.9 V *vs*. RHE. (**b**) Chronoamperometric tests for commercial Pt/C measured at 0.6 V *vs*. RHE.

**Figure S19. The EDS mapping results of PtPdNiCuMnAu HENCs after ADT from various random areas.**

**Figure S20**. (**a**) HAADF-STEM image of PtPdNiCu NCs after ADT, (**b**) corresponding elemental composition.

**Figure S21.** Changes in relative atomic ratio (*vs*. Pt) of each component before and after ADT for **(a)** PtPdNiCu NCs, **(b)** PtPdNiCuMn NCs and **(c)** PtPdNiCuMnAu HENCs, as determined by ICP-OES/MS.

**Figure S22.** The top and side view of (**a**) Pt_26.8_Pd_8.7_Ni14.3Cu_46.4_Mn_2.9_Au_0.9_, (**b**) Pt_27.5_Pd_9.7_Ni_15.1_Cu_43.3_Mn_3.6_Au_0.8_, (**c**) Pt_28.6_Pd_10.8_Ni_6.3_Cu_50.2_Mn_3.2_Au_0.9_ and (**d**) Pt_28.9_Pd_12.2_Ni_6.0_Cu_49.3_Mn_2.8_Au_0.8_ (111) surface models.

**Figure S23.** I-V polarization and power density plots of MEA with commercial Pt/C as the anode catalyst and PtPdNiCu NCs as the cathode catalyst.

**6. Tables**

**Table S1.** The atomic ratio of PtPdNiCuMnAu nanocrystals obtained from the drip strategy and one-pot strategy.

|  | PtPdNiCuMnAu nanocrystals obtained from the drip strategy | PtPdNiCuMnAu nanocrystals obtained from the one-pot strategy |
| --- | --- | --- |
| Pt | 11.1% | 8.46% |
| Pd | 6.81% | 34.1% |
| Ni | 33.9% | 0.49% |
| Cu | 45.9% | 53.9% |
| Mn | 2.18% | 2.93% |
| Au | 0.11% | 0.12% |

**Table S2.** The extracted data from the XRD patterns

| Catalysts | Diffraction peak | 2θ/ ° | θ/ rad | FWHM/ ° | β/ rad |
| --- | --- | --- | --- | --- | --- |
| PtPdNiCuMnAu HENCs | (111) | 41.0 | 0.358 | 1.10 | 0.0192 |
|  | (200) | 47.5 | 0.414 | 1.35 | 0.0236 |
| PtPdNiCu NCs | (111) | 41.4 | 0.361 | 0.85 | 0.0148 |
|  | (200) | 48.0 | 0.419 | 1.00 | 0.0175 |

The Williamson-Hall equation is expressed as:

$$\text{β∙}\cos\theta=K\lambda/D+4\varepsilon\cdot\sin\theta$$

β is the physical FWHM (in radians) after instrumental broadening correction. *θ* is the Bragg angle. *K* is the form factor, usually taken as 0.9. λ is the X-ray wavelength (Cu Kα, λ=0.15406 nm).

**Table S3.** Electronegativity of each element.

| **Element** | Pt | Pd | Ni | Cu | Mn | Au |
| --- | --- | --- | --- | --- | --- | --- |
| **Electronegativity** | 2.28 | 2.20 | 1.91 | 1.90 | 1.55 | 2.54 |

**Table S4.** The comparison of mass activity for recently reported studies.

| Catalysts | Mass activity | Electrolyte type | References |
| --- | --- | --- | --- |
| PtPdNiCuMnAu HENCs | 1.70 A/mg_Pt_ | 0.1 M KOH | This work |
|  | 1.32 A/mg_PGM_ |  |  |
|  | 1.26 A/mg_Pt_ | 0.1 M HClO_4_ |  |
|  | 0.98 A/mg_PGM_ |  |  |
| PtPdNiCu NCs | 0.61 A/mg_Pt_ | 0.1 M KOH |  |
|  | 0.51 A/mg_PGM_ |  |  |
|  | 0.77 A/mg_Pt_ | 0.1 M HClO_4_ |  |
|  | 0.65 A/mg_PGM_ |  |  |
| Pd@PtIrFe-28 NWs | 1.67 A/mg_PGM_ | 0.1 M HClO_4_ | *Fuel*, 2025, 389: 134581 |
| Octahedral PtCu_1.60_/C | 1.42 A/mg_Pt_ | 0.1 M HClO_4_ | *Nano Research*, 2023, 16(2): 2252-2258 |
| PtCoV EPNF | 1.40 A/mg_Pt_ | 0.1 M HClO_4_ | *Advanced Materials*, 2025, 37(34): 2502457 |
| L1_0_-PtCoCr/C | 1.27 A/mg_Pt_ | 0.1 M HClO_4_ | *Nature Communications*, 2025, 16(1): 4895 |
| Pd-Pt MNs | 1.19 A/mg_PGM_ | 0.1 M KOH | *Advanced Functional Materials*, 2025: e22126 |
| L1_0_-PtZn/Pt | 1.18 A/mg_Pt_ | 0.1 M HClO_4_ | *Advanced Functional Materials*, 2025: e13851 |
| Pt-Pd-Ni Nanocages | 1.17 A/mg_Pt_ | 0.1 M HClO_4_ | *Nano Energy*, 2019, 64: 103890 |
| PtNiCo Nanocages | 1.03 A/mg_Pt_ | 0.1 M HClO_4_ | *Journal of Materials Chemistry A*, 2021, 9(41): 23444-23450 |
| Pd_3_Gd/C-600 | 0.94 A/mg_Pd_ | 0.1 M KOH | *Advanced Functional Materials*, 2025: e14759 |
| PdMo MNVs/C | 0.92 A/mg_metal_ | 0.1 M KOH | *Advanced Materials*, 2025, 37(38): 2508055 |
| PtPdCu MEANPTs | 0.823 A/mg_PGM_ | 0.1 M KOH | *Journal of Alloys and Compounds*, 2024, 1008: 176859 |
| H-PtCo NPs | 0.69 A/mg_Pt_ | 0.1 M HClO_4_ | *Journal of Energy Chemistry*, 2025, 107: 713-721 |
| Pt-HEI@Pt/C | 0.65 A/mg_Pt_ | 0.1 M HClO_4_ | *Advanced Functional Materials*, 2025: 2503628 |
| L1_0_-PtFe@FeNC | 0.592 A/mg_Pt_ | 0.1 M HClO_4_ | *Chinese Journal of Catalysis*, 2025, 75: 125-136 |
| Pt-Ir-Pd Nanocages | 0.52 A/mg_PGM_ | 0.1 M HClO_4_ | *Advanced Energy Materials*, 2020, 10(16): 1904114 |
| O-PtCu | 0.48 A/mg_Pt_ | 0.1 M HClO_4_ | *Nano Research*, 2024, 17(8): 7001-7012 |
| PtCu Nanocages | 0.36 A/mg_Pt_ | 0.1 M KOH | *International Journal of Hydrogen Energy*, 2023, 48(43): 16286-16293 |
| SOCT CuPt Nanocages | 0.30 A/mg_Pt_ | 0.1 M HClO_4_ | *ACS Sustainable Chemistry & Engineering*, 2020, 8(28): 10544-10553 |
| Pd_17_@Pt_80_Mn_3_ NWs | 0.25 A/mg_Pt_ | 0.1 M KOH | *Materials Today Energy*, 2025, 53: 102008 |
| Pt@mPt CBNs | 0.21 A/mg_Pt_ | 0.1 M HClO_4_ | *ACS Sustainable Chemistry & Engineering*, 2018, 6(9): 11768-11774 |

**Table S5.** The atomic ratio of PtPdNiCu nanocages obtained from different etching conditions.

| Etching conditions | | Atomic ratio | | | | configurational entropy |
| --- | --- | --- | --- | --- | --- | --- |
| HAc/ mL | HNO_3_/ mL | Pt | Pd | Ni | Cu |  |
| 2 | 0.5 | 11% | 7% | 20% | 62% | 1.05*R* |
| 2 | 1.0 | 19% | 10% | 15% | 56% | 1.15*R* |
| 2 | 2.0 | 29% | 10% | 20% | 41% | 1.27*R* |

**Table S6.** The elemental composition of each catalyst (at%).

|  | Pt | Pd | Ni | Cu | Mn | Au | Configurational entropy |
| --- | --- | --- | --- | --- | --- | --- | --- |
| PtNiCu NCs | 22.2 |  | 4.4 | 73.4 |  |  | 0.70*R* |
| PdNiCu NCs |  | 33.0 | 8.0 | 59.0 |  |  | 0.88*R* |
| PtPdNiCu NCs | 29.3 | 9.6 | 20.0 | 41.1 |  |  | 1.27*R* |
| PtPdNiCu NCs  after ADT testing | 19.1 | 6.7 | 15.3 | 58.9 |  |  | 1.10*R* |
| PtPdNiCuMnAu HENCs | 23.0 | 12.6 | 15.6 | 44.2 | 3.3 | 1.3 | 1.42*R* |
| PtPdNiCuMnAu HENCs after ADT testing | 26.4 | 9.2 | 12.1 | 49.1 | 1.8 | 1.4 | 1.31*R* |
| PtPdNiCuMnAu HENCs  with 1.0 μmol Au^3+^ | 23.8 | 12.2 | 15.8 | 43.0 | 3.5 | 1.7 | 1.44*R* |
| PtPdNiCuMnAu HENCs  with 1.5 μmol Au^3+^ | 23.6 | 13.0 | 15.2 | 42.9 | 3.4 | 1.9 | 1.45*R* |
| PtPdNiCuAu HENCs | 12.3 | 6.7 | 13.6 | 66.3 |  | 1.1 | 1.03*R* |
| PtPdNiCuMnAu HENCs  with 28 μmol Mn^3+^ | 26.8 | 8.7 | 14.3 | 46.4 | 2.9 | 0.9 | 1.34*R* |
| PtPdNiCuMnAu HENCs  with 42 μmol Mn^3+^ | 27.5 | 9.7 | 15.1 | 43.3 | 3.6 | 0.8 | 1.39*R* |
| PtPdNiCuMnAu HENCs  with 56 μmol Mn^3+^ | 28.6 | 10.8 | 6.3 | 50.2 | 3.2 | 0.9 | 1.27*R* |
| PtPdNiCuMnAu HENCs  with 70 μmol Mn^3+^ | 28.9 | 12.2 | 6.0 | 49.3 | 2.8 | 0.8 | 1.27*R* |

**Table S7.** The *E*_1/2_ comparison of each catalyst in this work.

| **Catalysts** | ***E*_1/2_/ V vs. RHE** |
| --- | --- |
| PtNiCu NCs | 0.888 |
| PdNiCu NCs | 0.733 |
| PtPdNiCu NCs | 0.897 |
| PtPdNiCuMnAu HENCs with 1.0 μmol Au^3+^ | 0.857 |
| PtPdNiCuMnAu HENCs with 1.5 μmol Au^3+^ | 0.849 |
| PtPdNiCuAu HENCs | 0.882 |
| PtPdNiCuMnAu HENCs with 28 μmol Mn^3+^ | 0.911 |
| PtPdNiCuMnAu HENCs with 42 μmol Mn^3+^ | 0.902 |
| PtPdNiCuMnAu HENCs with 56 μmol Mn^3+^ | 0.898 |
| PtPdNiCuMnAu HENCs with 70 μmol Mn^3+^ | 0.889 |

**Table S8.** Content of each metal salt in the precursor solutions

|  | Pt(acac)_2_  (μmol) | Pd(acac)_2_  (μmol) | Ni(acac)_2_  (μmol) | CuCl_2_ 2H_2_O  (μmol) | Mn(acac)_3_  (μmol) | HAuCl_4_  (μmol) |
| --- | --- | --- | --- | --- | --- | --- |
| PtNiCu NCs | 22.9 | -- | 40.0 | 80.0 | -- | -- |
| PdNiCu NCs | -- | 23.0 | 40.0 | 80.0 | -- | -- |
| PtPdNiCu NCs | 19.7 | 3.2 | 40.0 | 80.0 | -- | -- |
| PtPdNiCuMnAu HENCs | 19.7 | 3.2 | 40.0 | 80.0 | 14 | 0.5 |
| PtPdNiCuMnAu HENCs  with 1.0 μmol Au^3+^ | 19.7 | 3.2 | 40.0 | 80.0 | 14 | 1.0 |
| PtPdNiCuMnAu HENCs  with 1.5 μmol Au^3+^ | 19.7 | 3.2 | 40.0 | 80.0 | 14 | 1.5 |
| PtPdNiCuAu HENCs | 19.7 | 3.2 | 40.0 | 80.0 | -- | 0.5 |
| PtPdNiCuMnAu HENCs  with 28 μmol Mn^3+^ | 19.7 | 3.2 | 40.0 | 80.0 | 28 | 0.5 |
| PtPdNiCuMnAu HENCs  with 42 μmol Mn^3+^ | 19.7 | 3.2 | 40.0 | 80.0 | 42 | 0.5 |
| PtPdNiCuMnAu HENCs  with 56 μmol Mn^3+^ | 19.7 | 3.2 | 40.0 | 80.0 | 56 | 0.5 |
| PtPdNiCuMnAu HENCs  with 70 μmol Mn^3+^ | 19.7 | 3.2 | 40.0 | 80.0 | 70 | 0.5 |

**Table S9.** ICP-OES/MS results for PtPdNiCu NCs, PtPdNiCuMn NCs and PtPdNiCuMnAu HENCs before and after ADT.

|  |  | Before ADT | After ADT |
| --- | --- | --- | --- |
| PtPdNiCu NCs | Pt | 2.2432 mg/L | 1.8573 mg/L |
|  | Pd | 0.6188 mg/L | 0.5172 mg/L |
|  | Ni | 0.3328 mg/L | 0.2614 mg/L |
|  | Cu | 1.4524 mg/L | 1.0695 mg/L |
| PtPdNiCuMn NCs | Pt | 2.2143 mg/L | 1.7614 mg/L |
|  | Pd | 0.8363 mg/L | 0.7012 mg/L |
|  | Ni | 0.4204 mg/L | 0.2338 mg/L |
|  | Cu | 1.6349 mg/L | 0.8667 mg/L |
|  | Mn | 8.7868 μg/L | 3.1856 μg/L |
| PtPdNiCuMnAu HENCs | Pt | 3.1912 mg/L | 3.1324 mg/L |
|  | Pd | 0.9575mg/L | 0.9398 mg/L |
|  | Ni | 0.6528 mg/L | 0.5937 mg/L |
|  | Cu | 1.9960 mg/L | 1.7857 mg/L |
|  | Mn | 0.1258 mg/L | 0.0970 mg/L |
|  | Au | 0.1933 mg/L | 0.1771 mg/L |
